# Supplementary material for: Chromatic micromaps in primary visual cortex
Source: Nat Commun. 2021 Apr 19;12:2315. doi: 10.1038/s41467-021-22488-3 (PMC8055908; doi:10.1038/s41467-021-22488-3)
Supplement: Supplementary file 1 — Supplementary Information [file 41467_2021_22488_MOESM1_ESM.pdf]

Chromatic micromaps in primary visual cortex
Supplementary Information

Chatterjee et al.

Supplementary Figures

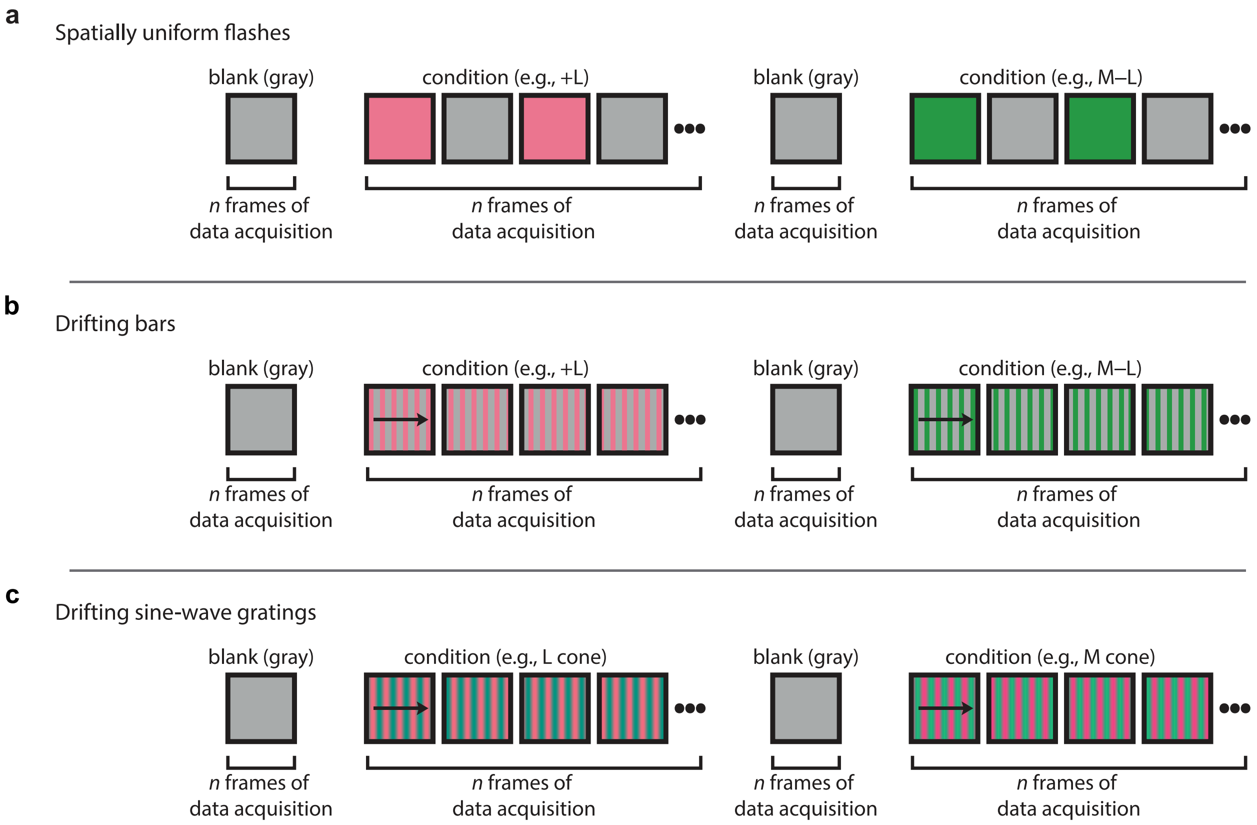

Supplementary Figure 1 | Schematic representation of color stimuli. An identical set of color conditions was presented as either **a**, spatially unstructured (uniform) flashes, or **b**, spatially structured drifting bars, with blanks and conditions of equal duration. **c** A separate set of color conditions was presented as drifting sinusoidal gratings, with blanks and conditions of equal duration.

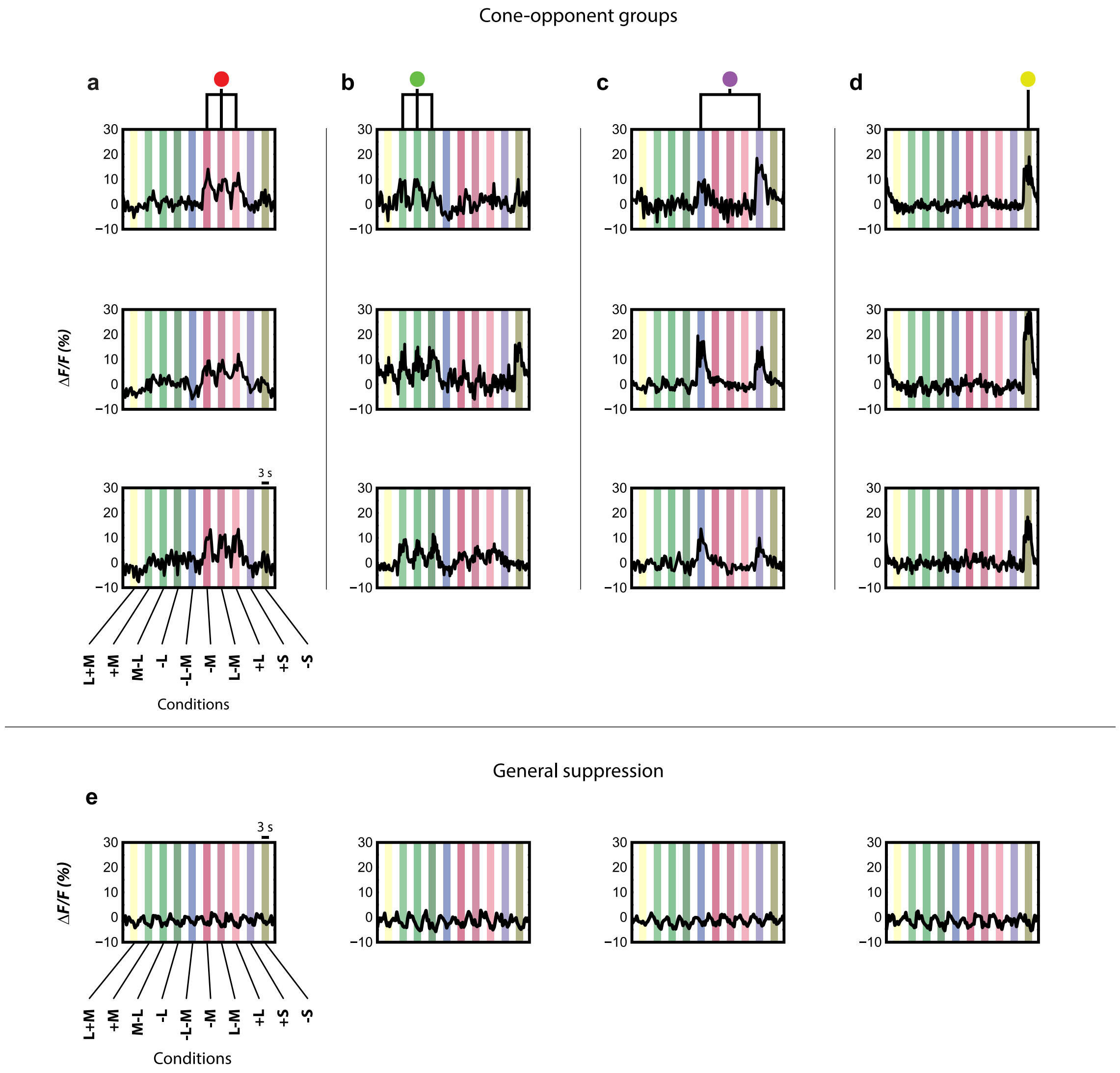

Supplementary Figure 2 | Responses to spatially uniform color flashes. **a** Time courses for three cells exhibiting L–M cone opponency, averaged over all repeats. Colored epochs denote stimulation periods (epoch color approximating stimulus color), and interleaved white epochs are blanks, with the last blank being redrawn from the first. These three cells responded best to +L, L–M, or –M conditions, defined as class 'red'. The dots above the conditions which define a class are the same color as cell markers in Figs. 2 and 3. There were 829 red cells in our total population of 3,365 cells responsive to uniform stimuli (24.6%). **b** Three M–L cells, same conventions as in **a**. For these, the preferred responses are to +M, M–L, or –L, defined as class 'green' (778 cells; 23.1%). Included is an example of a border cell (second panel) found between adjoining M–L and –S subdomains, showing that L/M and S axes can be combined in individual cells. **c** Three S–(L+M) cells with preferred responses to the +S or –L–M conditions, defined as class 'blue'

(714 cells; 21.2%). **d** Three –S cells, defined as class 'yellow' (980 cells; 29.1%). Cells that preferred non-opponent L+M stimuli, including an L+M+S condition run in one animal (the 'luminance' class), were rare in our population (64 cells; 1.9%) and are not shown here. Cell numbers are from 18 FOVs, shown in Supplementary Table 1. **e** Four examples of cells that were suppressed by all spatially uniform stimuli (significantly responsive cells with $\Delta F/F$ < response to blank for each condition).

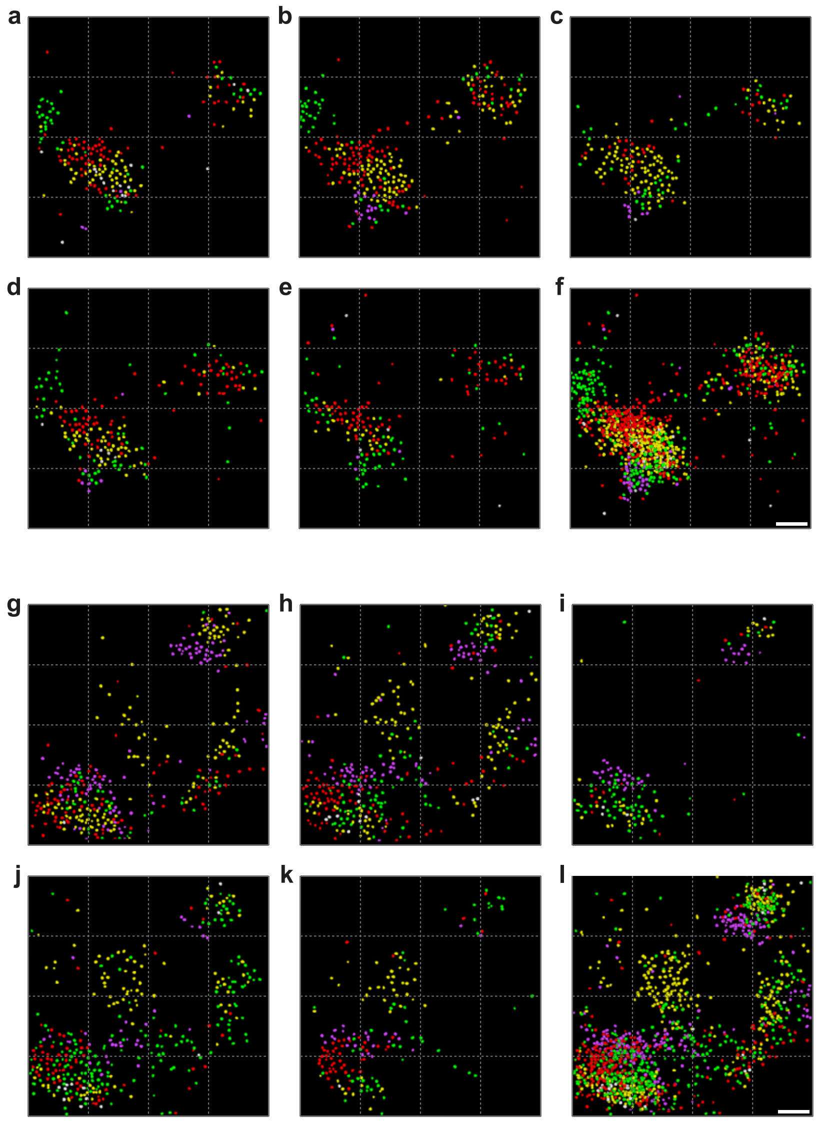

Supplementary Figure 3 | Micromaps across multiple depths. Data from two animals with complete micromaps and 5 or more depths imaged in a column. **a-e** Cell-based maps from first animal (individual planes for Fig. 2g). Depths: 200, 230, 260, 300, 320 μm; counts of responsive cells (segmented cells): 192 (1,267), 270 (1,186), 168 (703), 193 (1,047), 165 (1,018), respectively. **f** combined map. **g-k** Cell-based maps from second animal. Depths: 80, 100, 120, 140, 220 μm; counts of responsive cells (segmented cells): 361 (1,887), 376 (1,857), 136 (1,789), 368 (1,814), 168 (1,700), respectively. **l** combined map. Scale bars, 100 μm.

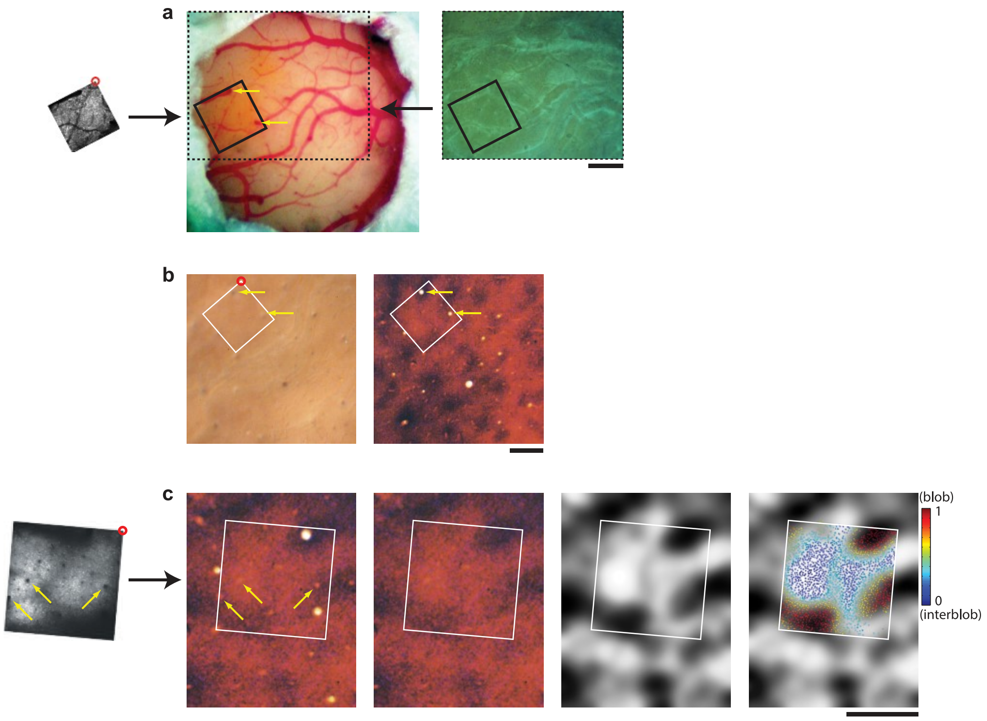

Supplementary Figure 4 | Alignment of two-photon imaging with cytochrome oxidase histology. **a** Initial gross alignment relied on surface vasculature as fiduciary markers. The region of cortex containing the functionally imaged FOV was located by comparing the vessel shadows in a two-photon surface image (small image, left) with a photograph of the full vasculature pattern taken *in vivo* (left panel; position of FOV shown with solid black square). Note the 'X' of fine vessels that marks the center of the FOV. After perfusion (right panel), the tissue was realigned using surface vessels visible under a dissecting microscope (seen here as autofluorescence with a FITC filter; alignment represented by dashed black rectangle). **b** Prominent radial vessels (arrows) served as a check of gross alignment between the unstained tissue (left panel), the cytochrome stained tissue (right panel), and the *in vivo* vasculature pattern in **a**. The position of the FOV is overlaid as a white box. **c** Fine alignment was achieved by comparing the much smaller radial vasculature (arrows) seen in a deeper two-photon image (small image, left) and the stained tissue (first panel). Following alignment, the tissue image was processed to remove vessel artifacts (second panel), converted to grayscale, bandpass filtered and smoothed (third panel), and used to define cytochrome oxidase values (from 0 to 1, lightest to darkest) for each identified cell in the two-photon imaged plane (fourth panel). Scale bars, 500 µm. Slight changes in rotation of FOV between **a-c** can be followed using the small red circle in the corner of the FOV images.

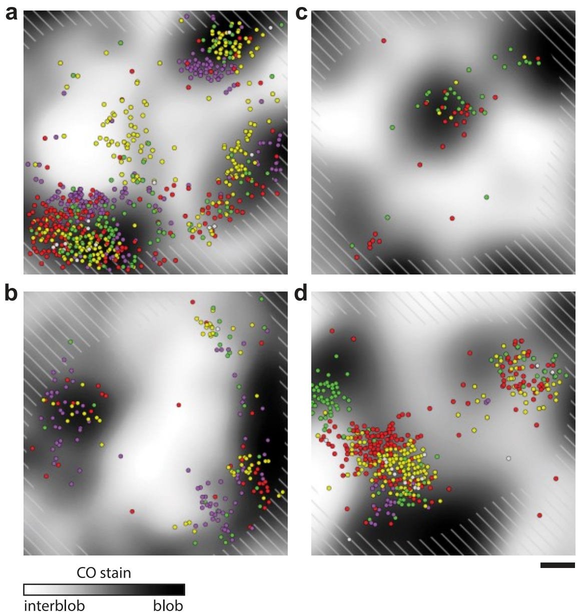

Supplementary Figure 5 | Color micromap columns align with cytochrome oxidase blobs. a-d Representative cell-based response maps from four animals. Each functional map is drawn on a grayscale representation of its cytochrome oxidase histology, same conventions as in Fig. 3a. Virtually all color micromap columns were in register with areas of high cytochrome oxidase staining, but our sample of color maps also showed heterogeneity. There were imaged regions (**a** and **d**) that had blobs with complete color micromaps: +S, –S, and both classes of red/green neurons in a single large cluster. Other regions had blobs that were biased toward one color axis, either strongly (red/green in **c**) or weakly (blue/yellow in **b**). Finally, some color patches extended from blobs into areas of moderate cytochrome oxidase staining (**a** and **d**), one of which was a clear chromatic bridge between blobs of different ocular dominance[1] (**d**, left cluster). **a** is redrawn from Fig. 3a. **d** shows the blob pattern for the example in Fig. 2. Cell maps taken from depths of 195, 215 (**a**), 180 (**b**), 200, 225 (**c**), and 200, 230 μm (**d**). Scale bar, 100 μm.

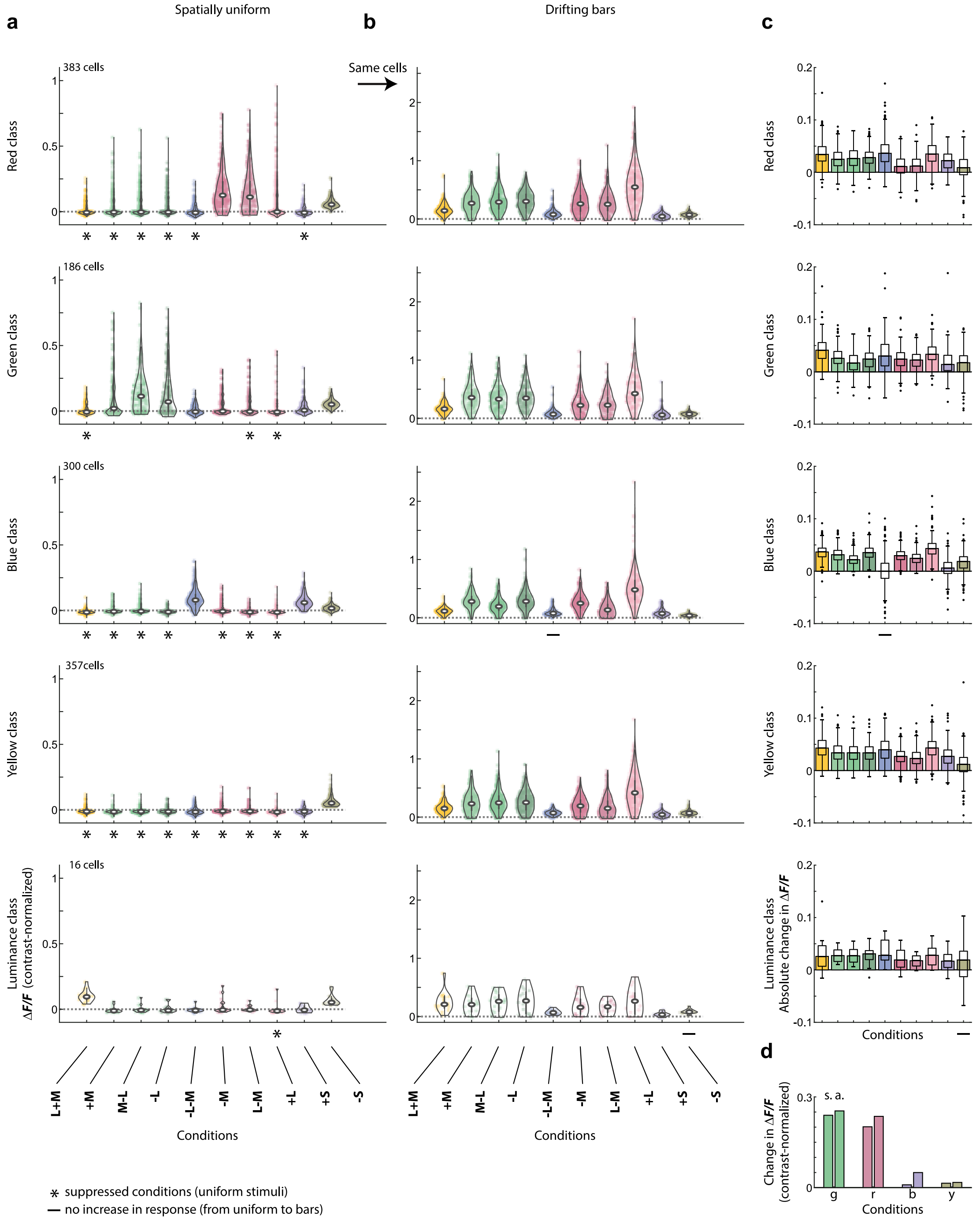

Supplementary Figure 6 | Responses to spatially uniform and drifting bar color conditions. Data from 12 FOVs tested with the full set of 10 color conditions presented as both spatially structured and

unstructured stimuli (1,242 coactive cells significantly responsive to both stimulus types, out of 14,576 segmented; same population as in Fig. 4b-d).

a Distributions of $\Delta F/F$ responses, normalized by stimulus contrast (see Methods), to color conditions presented as spatially uniform flashes. Responses to all conditions are plotted for each cell in a class (e.g., with 383 cells in the red class, each of the 10 distributions in the top panel contains 383 data points). Asterisks mark conditions for which the median response distribution was significantly below baseline ($P < 0.01$, Wilcoxon signed-rank test on unnormalized data, Holm-Bonferroni correction for multiple comparisons; $n_{\mathrm{red}} = 383$, $n_{\mathrm{green}} = 186$, $n_{\mathrm{blue}} = 300$, $n_{\mathrm{yellow}} = 357$, $n_{\mathrm{luminance}} = 16$). Generally, conditions on the same color axis (red/green or blue/yellow) but opponent to the preferred conditions of a class evoked significant suppression in that population, as with the green condition responses (M, M–L, –L) in the red class (which respond best to L, L–M, –M; top panel), and vice versa (second panel). There was also significant inter-axis suppression, most pronounced in the blue (S, –L–M) and yellow (–S) classes in which the distributions of responses to all red/green conditions were significantly below baseline (third and fourth panels). The most striking inter- and intra-axis suppression was found in the yellow class, in which every condition except the preferred –S evoked a distribution of responses significantly below baseline. Further, the –S condition did not suppress responses in any of the other classes. Finally, the luminance condition (L+M) suppressed responses in all classes except for the 16 cells (1.3% of 1,242 coactive cells) for which that was the best condition.

b Same cells and color conditions as in **a**, but with stimuli presented as drifting bars. The addition of spatial structure caused significant increases in response to almost all conditions in all cell classes (vertical axes have different scales in **a** and **b**). Only 2 of the 50 condition/class pairs resulted in no significant increase in median response (indicated with a bar below those distributions; $P < 0.01$, Wilcoxon signed-rank test on unnormalized data). The largest increases were seen with red/green conditions, to the extent that previously distinct response patterns that defined a class (e.g., the blue class in the third panel of **a**, with dominant +S and –L–M responses) become far less clear with bars (third panel of **b**). Class-specific suppression patterns disappeared completely with bars, further eroding differences in response profiles between classes. Thus, as a population, each class became more similar in its distribution of responses. However, individual cells

often showed the same distinct response profiles to bars that were used to define color classes with uniform stimuli (e.g., traces in Supplementary Fig. 9f).

c Boxplots showing the absolute (unnormalized) change in $\Delta F/F$ between uniform and bar conditions, for each cell in each class. Same order of conditions as in **a**.

d Contrast-normalized summary of data in **c**. Stimuli have been grouped into green (M, M–L, –L), red (L, L–M, –M), blue (S, –L–M), and yellow (–S) conditions, and the height of each bar indicates the median change in response to all stimuli in the group. The first bar of each group, marked (s)ame, shows the change in response to that group of conditions seen only in cells of that class (e.g., the first bar of the plot represents green cells responding to green conditions). Each of these is significantly different from the other, except the red and green groups ($P < 0.01$, Kruskal-Wallis test, post-hoc comparisons using Tukey HSD). The second bar of each group, marked (a)ll, shows the change in response to that group of conditions seen in all the cells. Each group is significantly different from the other ($P < 0.01$, Kruskal-Wallis test, post-hoc comparisons using Tukey HSD). The increase in response to bars was dominated by the increase to red/green conditions. Very little of the overall increase was driven by blue/yellow conditions.

Violin plots in **a**, **b** show medians (white ovals) and interquartile ranges (IQR; thick center lines). Boxplots in **c** show medians (heights of colored bars), IQR (boxes), data 1.5 × IQR above or below 75th or 25th percentiles (whiskers), and outliers beyond this range (small points). All plots colored to approximate color of stimulus conditions.

Source data are provided as a Source Data file.

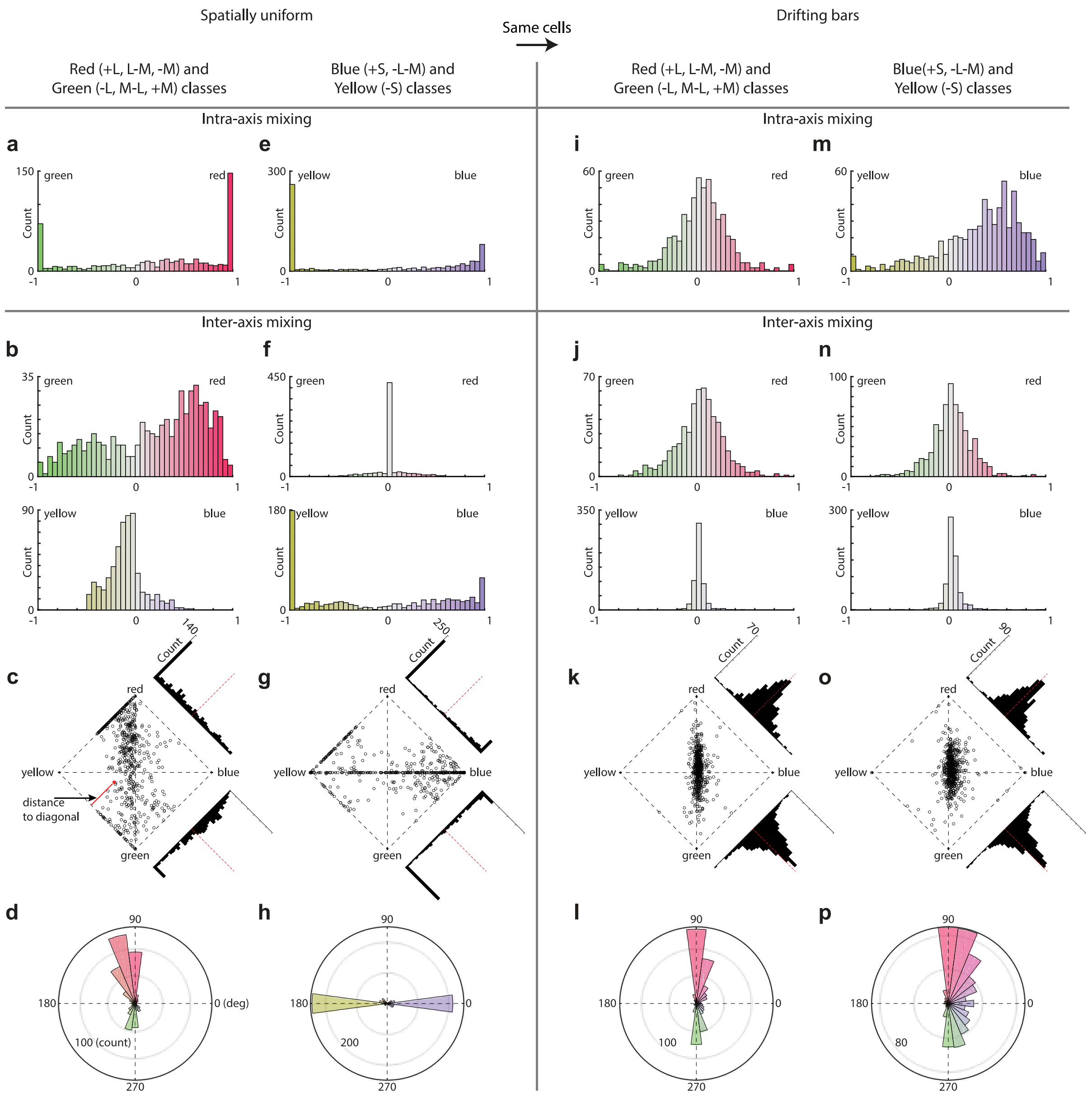

Supplementary Figure 7 | Intra- and inter-axis contributions to color responses.

a-d Color tuning of cells in the red and green classes in response to spatially uniform stimuli (same population as in Supplementary Fig. 6a, top two panels; $n_{\text{red}} = 383$, $n_{\text{green}} = 186$).

a Cells in a given color class responded best to some combination of the opponent conditions defining the class, but some also responded to conditions from the opposite end of the class's dominant axis, like the smaller positive responses to the green conditions (M, M–L, –L) seen in the red (L, L–M, –M) cell

population (Supplementary Fig. 6a, top panel). This motivated the construction of a continuous-valued measure of intra-axis mixing for each cell as a way of quantifying response purity (e.g., how exclusively red a red cell is). An intra-axis index was calculated by summing each cell's contrast-normalized, positive responses to all red conditions and subtracting the responses to all green conditions, normalized by the total response to all red and green conditions (Supplementary Equation 1). Suppressed responses were set to zero. The distribution of this value (histogram in **a**) has distinct peaks at +1 and -1, indicating mostly pure responses to red or green stimuli from red- or green-class cells, respectively, when stimulated with spatially uniform conditions. Impure or mixed responses (more closely matched responses to red and green conditions) were rare, with very few cells clustered near 0.

$$\text{intraRG} = \frac{\sum \text{red} - \sum \text{green}}{\sum \text{red} + \sum \text{green}} \quad \text{(Supplementary Equation 1)}$$

b The *inter*-axis contributions of both red/green and blue (+S, –L–M)/yellow (–S) responses were calculated in a similar way, except the response to conditions along the red/green (Supplementary Equation 2) and blue/yellow (Supplementary Equation 3) axes were normalized by the summed responses to all color conditions (luminance condition excluded), giving the relative contribution of each axis to the total response of a cell. The distributions are shown separately (red/green, upper histogram; blue/yellow, lower histogram). Values near +1 or -1 indicate a pure response to a single class of conditions, but the shift toward 0 in the upper histogram signifies some intra-axis (blue/yellow) contributions to these cells. The distribution clusters around 0 in the lower histogram because the net contribution of blue/yellow responses is generally small for red/green cells.

$$\text{interRG} = \frac{\sum \text{red} - \sum \text{green}}{\sum \text{red} + \sum \text{green} + \sum \text{blue} + \sum \text{yellow}} \quad \text{(Supplementary Equation 2)}$$

$$\text{interBY} = \frac{\sum \text{blue} - \sum \text{yellow}}{\sum \text{red} + \sum \text{green} + \sum \text{blue} + \sum \text{yellow}} \quad \text{(Supplementary Equation 3)}$$

c The red/green and blue/yellow responses of each cell were combined into a single plot, with the vertical (red/green) coordinate given by Supplementary Equation 2 (interRG) and the horizontal (blue/yellow) coordinate by Supplementary Equation 3 (interBY). Cells at the ends of axes had pure responses to a single class of conditions, without intra- or inter-axis mixing. Cells along the dashed diagonals had pure intra-axis responses along both axes (e.g., cells on the $y = x + 1$ diagonal gave red responses with no green, yellow responses with no blue). Marginal histograms show the preponderance of these cells (centers marked with dashed red lines). The greater a cell's distance from its closest diagonal, the less pure the contributions from one or both axes (i.e., more intra-axis mixing).

d Polar histogram summary of data in **c**. The preferred color direction θ of each cell is the direction of the vector sum of red/green (interRG) and blue/yellow (interBY) responses (i.e., the direction of the vector from the origin to the cell's location on the scatterplot).

e-h Same analyses as **a-d**, but for cells classified as blue/yellow (same population as in Supplementary Fig. 6a, third and fourth panels; $n_{\text{blue}} = 300$, $n_{\text{yellow}} = 357$). Intra-axis mixing was quantified using Supplementary Equation 1, but with Σred and Σgreen replaced by Σblue and Σyellow, respectively. Supplementary Equations 2 and 3 did not change. **i-l** Same analyses as **a-d**, same cells, but with responses to drifting bars. **m-p** Same analyses as **e-h**, same cells, but with responses to drifting bars. When going from unstructured to structured stimuli, there was a shift away from very pure responses along both axes — cells clustered along the diagonals in **c** and **g** — to responses with far more intra-axis mixing, with almost no cells along the diagonals in **k** and **o** (see Supplementary Fig. 8a).

The median red/green intra-axis index in response to drifting bars was 0.10 for cells in the red class and -0.04 for cells in green (**i**), which, while still significantly different from each other ($P = 2.8 \times 10^{-14}$, Wilcoxon rank sum test), were much closer than the wide separation in medians obtained with uniform stimuli (0.71 and -0.74 for red and green cells, respectively; $P = 4.8 \times 10^{-81}$, Wilcoxon rank sum test; panel **a**). Similarly, the median blue/yellow intra-axis index to drifting bars was 0.63 for cells in the blue class and 0.21 for cells in yellow (**m**), which were significantly different ($P = 2.4 \times 10^{-50}$, Wilcoxon rank sum test) but showed a pronounced shift toward the blue (positive) end of the axis, compared to the median indices obtained with uniform stimuli (0.81 and -1; $P < 10^{-100}$, Wilcoxon rank sum test; panel **e**). Note that Supplementary Equation 3 likely underestimates the yellow (–S) contribution to overall color tuning, since two conditions are summed for the blue term and one for yellow. However, using the mean, instead, underestimated contributions from classes with multiple conditions, since one condition often evoked a much larger response than the others. The general results of structured vs unstructured color tuning (Supplementary Fig. 8) and the shifts seen here did not depend on which form of the equation was used.

There was a weak negative correlation between red/green and blue/yellow inputs obtained with spatially uniform stimuli ($r = -0.08$, $n = 1{,}226$, $P = 0.007$; data from scatterplots **c** and **g**), supporting previous work[2] that suggested M- and S-cone responses were often aligned in sign. This correlation disappeared with bars ($r = 8 \times 10^{-4}$, $n = 1{,}226$, $P = 0.98$; data from scatterplots **k** and **o**).

Nonpolar histograms colored to reflect approximate color of stimuli, with saturation indicating response purity; 40 bins per axis. For polar histograms, intermediate colors between axes linearly interpolated in RGB space to approximate color tuning; bin width, 15 degrees.

Source data are provided as a Source Data file.

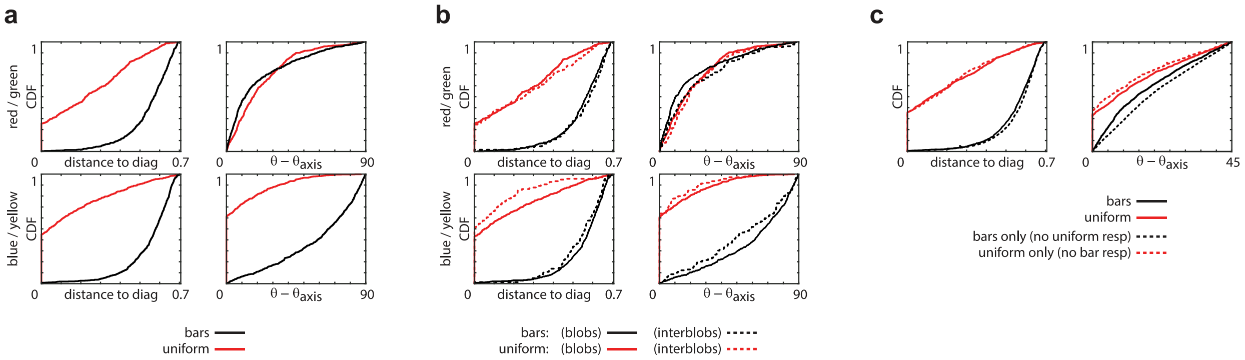

Supplementary Figure 8 | Color tuning in blobs, interblobs, and non-coactive cells.

a Cumulative distributions of color tuning metrics from Supplementary Fig. 7. Distance to diagonal (e.g., red line from red cell in Supplementary Fig. 7c) is a measure of how pure the red/green and blue/yellow contributions are. θ is a measure of preferred color direction (e.g., Supplementary Fig. 7d). Here we plot $\theta - \theta_{axis}$, the shortest angular distance between a cell's θ and the dominant axis of that cell's class (vertical axis for red/green cells, horizontal axis for blue/yellow), so smaller values of $\theta - \theta_{axis}$ signify smaller inter-axis contributions. Two-sample Kolmogorov-Smirnov tests used for all significance tests. Top left: distance to diagonal for red/green population in response to uniform vs bar stimuli ($n_{uniform} = 569$, $n_{bar} = 569$, $P < 10^{-100}$). Top right: $\theta - \theta_{axis}$ for red/green population in response to uniform vs bar stimuli ($P = 1.5 \times 10^{-6}$). Bottom left: same as top left, but for blue/yellow cells ($n_{uniform} = 657$, $n_{bar} = 657$, $P < 10^{-100}$). Bottom right: same as top right, but for blue/yellow cells ($P < 10^{-100}$).

b Same as **a**, but with populations divided into blob (CO values ≥ 0.5; solid lines) and interblob (CO < 0.5; dashed) cells. Top left: there was no difference in distance to diagonal for red/green cells in blobs vs interblobs to uniform stimuli ($n_{blobs} = 443$, $n_{interblobs} = 126$, $P = 0.29$) or to bars ($P = 0.36$). Top right: no difference in $\theta - \theta_{axis}$ for the same populations to uniform stimuli ($P = 0.34$) or to bars ($P = 0.027$). Bottom left: distance to diagonal was significantly different for blue/yellow cells in blobs vs interblobs to uniform stimuli ($n_{blobs} = 518$, $n_{interblobs} = 139$, $P = 1.8 \times 10^{-3}$), but not to bars ($P = 0.051$). Bottom right: no difference in $\theta - \theta_{axis}$ for the same populations to either uniform stimuli ($P = 0.52$) or to bars ($P = 0.010$).

c Comparison of tuning metrics between all coactive cells (analyzed in **a**, **b** and in Supplementary Figs. 6 and 7) and cells from the same FOVs that responded to either bar or uniform stimuli, but not both. Since we define color classes with respect to uniform-condition responses (thus excluding a classification of bars-

only cells), $\theta - \theta_{axis}$ here is the shortest angular distance to the closest axis, and values approaching 45 degrees indicate greater inter-axis mixing. Left: no significant difference in distance to diagonal between uniform-only cells and coactive cells in response to uniform stimuli ($n_{coactive}$ = 1,226, n_{only} = 695, P = 0.34); significant difference between bars-only cells and coactive cells in response to bars ($n_{coactive}$ = 1,226, n_{only} = 2,460, $P = 5.8 \times 10^{-4}$). Right: no difference in $\theta - \theta_{axis}$ between uniform-only cells and coactive cells ($n_{coactive}$ = 1,226, n_{only} = 695, P = 0.024); significant difference between bars-only cells and coactive cells ($n_{coactive}$ = 1,226, n_{only} = 2,460, $P = 2.2 \times 10^{-11}$).

Source data are provided as a Source Data file.

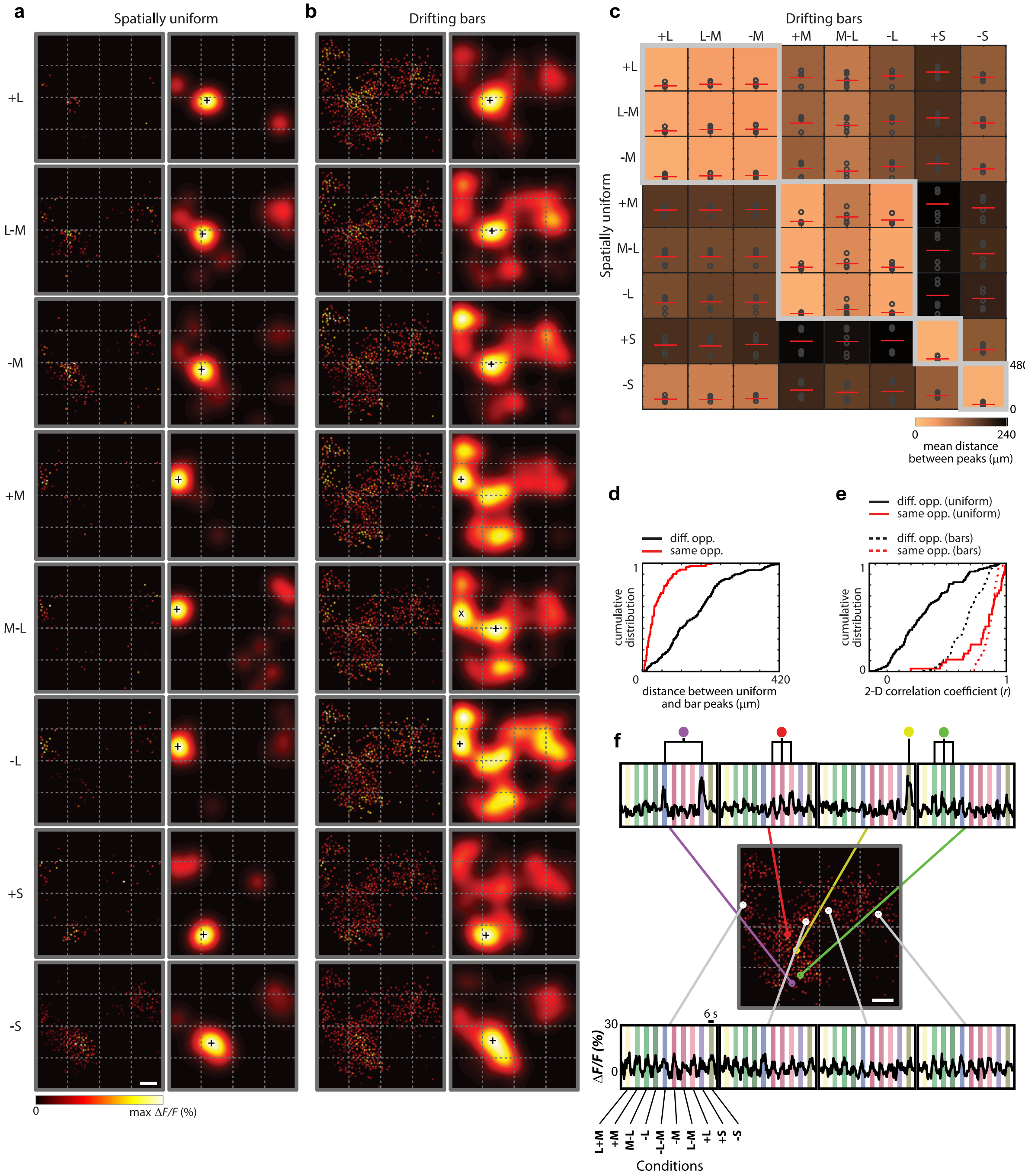

Supplementary Figure 9 | Spatial organization of responses to uniform and drifting bar stimuli.

a The eight left panels are cell-based response maps (Δ*F/F*) showing peak responses to spatially uniform color flashes (conditions indicated on far left) of significantly responsive cells, all from a single FOV. Cells responding best to stimuli from the red group (+L, L–M, –M) are largely segregated from cells preferring

the green group (+M, M–L, –L), and both clusters are distinct from +S and –S microdomains. The eight right panels are modified kernel density estimations (KDE) generated from the cell maps to better visualize hotspots and to clearly define local peaks (see Methods). We extracted all peaks with amplitude > 50% of the largest peak in each KDE map, which for spatially uniform stimuli usually gave only one peak in a color patch per stimulus condition (marked with a '+').

b The eight left panels show peak responses from the same FOV as in **a**, stimulated with the same set of colors, but in the form of drifting bars (best response for each cell selected from four directions of motion presented as separate runs). Responses to bars are generally larger in magnitude (see Supplementary Figs. 6 and 7), and activity spreads well beyond the chromatic subdomains obtained with uniform stimuli. In almost every case the largest extracted peak coincides with the peak found in the corresponding spatially uniform condition, showing that color preferences of the original domains are preserved in the expanded domains. Only one stimulus condition for this FOV gave a max peak which does not overlap with the peak seen with uniform stimuli (M–L, fifth row), but the second largest peak (marked with an 'x') does correspond with the peak in **a**, and its amplitude is almost identical (98% of max) to the largest.

c We quantified this correspondence between subdomains obtained with spatially uniform and bar stimuli by measuring the distance between the largest uniform peak and the nearest bar peak (with amplitude at least 50% of bar max) for all pairs of conditions for a given FOV. This was done for 6 FOVs from two animals having complete micromaps (well-defined KDE peaks for the three red, three green, +S and –S conditions). All pairwise distances are plotted in the matrix, with mean distance for each pair shown with both a red line and background colormap (colorbar, lower right). Boxes outlined in gray contain pairs belonging to same opponency group.

d Distribution of all bar/uniform peak distance pairs shown in **c**, from either the same opponency group (red) or different opponency groups (black). The two distributions are significantly different (n_{same} = 120 pairs, $n_{\text{different}}$ = 264 pairs; $P = 3.2 \times 10^{-31}$, two-sample Kolmogorov-Smirnov test).

e We quantified the increased overlap of microdomains observed with drifting bar stimuli (as seen in **b**) by calculating the two-dimensional correlation coefficient for each pair of KDE images from a given FOV (same FOVs as in **c**), for a given stimulus type (uniform, bars; see Methods). The distribution of coefficients for all uniform and bar pairs are shown (solid and dashed lines, respectively). As expected, images from the same opponency groups (red lines) are highly correlated (median $r_{\text{uniform}} = 0.86$, $r_{\text{bars}} = 0.88$), and the distributions are not significantly different ($n_{\text{uniform}} = n_{\text{bars}}$ = 36 pairs; $P = 0.18$, two-sample Kolmogorov-Smirnov test). The distributions diverge for different opponency groups (black lines). Images obtained with

uniform stimuli have much lower correlation coefficients (median $r = 0.28$), consistent with segregated, non-overlapping domains, while the significantly different bar distribution ($n_{uniform} = n_{bars} = 132$ pairs; $P = 1.4 \times 10^{-25}$, two-sample Kolmogorov-Smirnov test) remains shifted to the right (median $r = 0.68$), closer to those of the same-opponency groups.

f Combined best response map from the bar experiments in **b** with examples of single-cell time courses (conventions as in Supplementary Fig. 2; each trace from a single direction of motion). The top row of time courses represents cells with clear opponency. Some cells outside of color domains (bottom row) responded to most or all of the 10 color conditions, regardless of cone type or sign. There were 181 such cells (significantly responding to > 8 color conditions; Tukey HSD, $P < 0.05$) out of a population of 4,205 responsive cells from 12 FOVs (4.3%).

Scale bars, 100 µm (shared by **a**, **b**). Each response map in **a**, **b**, and **f** was drawn using the colormap shown at bottom of **a**, scaled to cover the colormap's full range. Max $\Delta F/F$ (%) for each condition (uniform; bars): +L (4.0; 10.1) –L (4.8; 7.4) +M (5.5; 8.1) –M (3.9; 7.6) +S (5.8; 10.7) –S (21.5; 17.7). For the map in **e**, max $\Delta F/F$ (%): 17.7.

Source data are provided as a Source Data file.

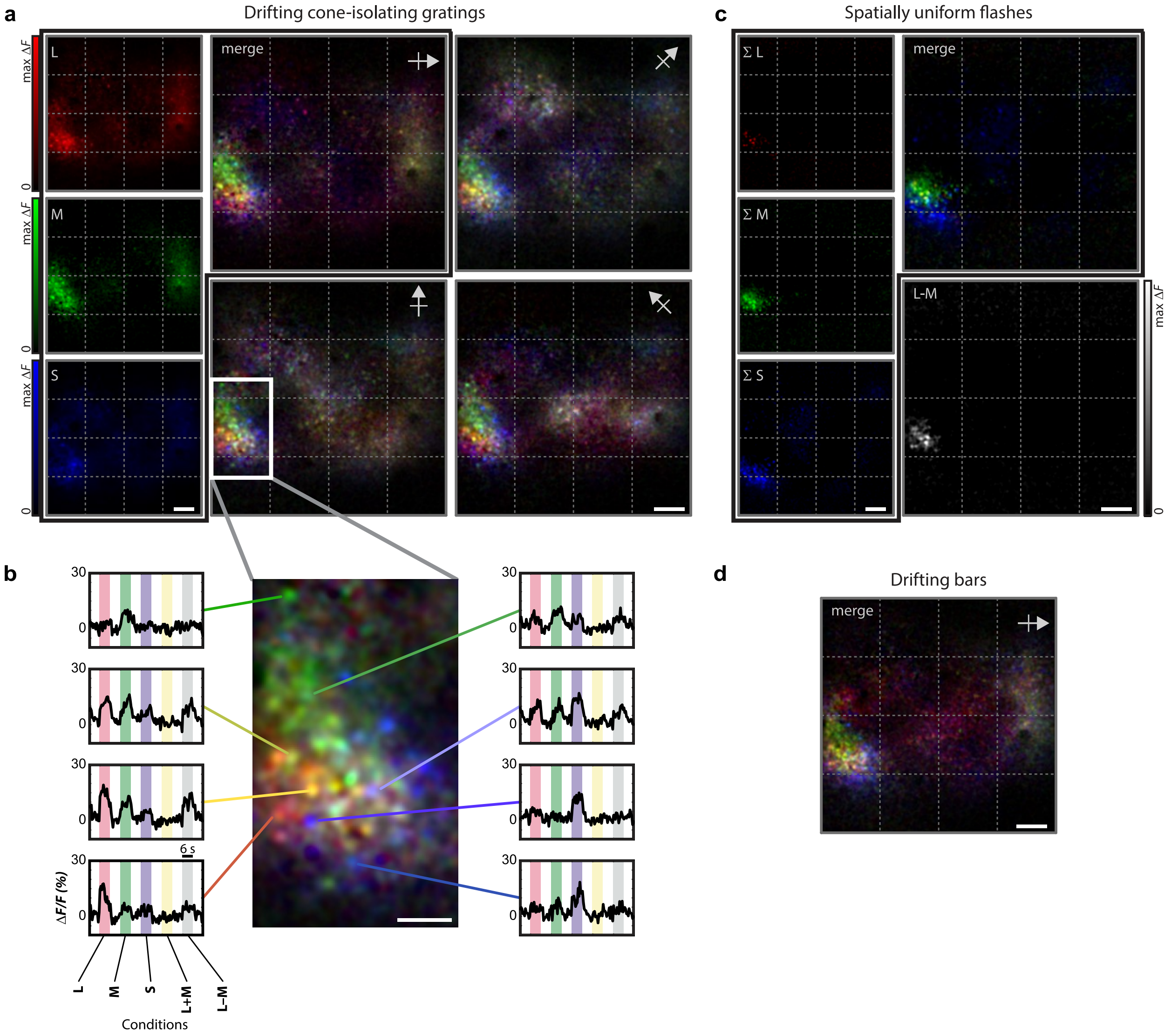

Supplementary Figure 10 | Comparison of functional clusters obtained with three different stimulus sets: drifting gratings, spatially uniform flashes, and drifting bars.

a The three far-left panels are response maps to L, M, and S cone-isolating sinusoidal gratings, drifting in one direction of motion (raw ΔF responses not normalized by cone contrast), merged in the larger connected panel to the immediate right. Three other directions of grating motion are also represented, indicated by inset arrows. The spatial response profiles consist primarily of active regions shifting with grating direction, which reflect interblob orientation domains. A conspicuous exception is found in the lower left of each map

(e.g., solid white box), where a bright cluster of blob cells exhibits a stable pattern of cone inputs persisting across all directions of motion.

b The magnified cluster, with representative $\Delta F/F$ time courses for individual cells, shows a distinct progression of cone responses from top to bottom, starting with an M-dominated region which smoothly transitions into a region of greater L input, which in turn transitions into a crescent of S-dominated cells on the lower and right edges of the cluster. There is considerable mixing of L/M and S inputs at the borders of subdomains. Further, the cells in the M- to L-dominated transition are all L/M opponent, as indicated by the last two conditions of each time course [in which L–M > L+M; cone contrasts matched at 8% for L and M in both conditions, (see Methods)]. Since gratings modulate cone activity both above (sine wave peak) and below (trough) mean activation (background gray), their response maps are a combination of ON and OFF responses for each cone type.

c To better visualize the differences in spatial clustering obtained with sign-ambiguous gratings and the spatially uniform stimulus set, which was sign-specific, the uniform maps were plotted as a sum of ON and OFF maps for each cone type (three left panels, merged in the connected panel on upper right). The chromatic organization in **c** shows far greater spatial segregation of L/M and S cone-dominated subdomains, as seen in every other uniform case (Supplementary Fig. 9a; ΔF maps in Figs. 1 and 2). Also, the size of the micromap is more constrained, with smaller individual subdomains and almost no activity outside the cluster. The L/M subdomain is purely +L/–M opponent, indicated by the L–M map (lower right).

d The visualization procedure of summing ON and OFF response maps was used with bar stimuli as well (one direction of motion). Although the color conditions of the bar stimuli were identical to those of the uniform stimuli, the resultant map fully recapitulates the pattern of cone preferences found with gratings in **a**.

Scale bars, 100 μm (**a**, **c**, **d**); 50 μm (**b**). Same FOV in each figure panel, 220 μm depth.

Supplementary Tables

FOV	Red<br>+L, L-M, -M	Green<br>M, M-L, -L	Blue<br>+S, -L-M	Yellow<br>-S	Luminance<br>L+M	Total<br>Responsive	Total<br>Segmented	Notes
1	34	32	260	122	3	451	1827	*a, b, c, f, i*
2	90	39	102	129	1	361	1887	*a, b, e, g, h*
3	89	76	79	118	14	376	1857	*a, b, e, g, h, j*
4	12	54	38	28	4	136	1789	*a, b, e*
5	64	170	32	90	12	368	1814	*a, b, e*
6	19	6	14	3	0	42	1695	*a, b,*
7	51	50	25	41	1	168	1700	*a, b, c, f, k, e*
8	70	45	4	58	15	192	1267	*a, b, c, d, f, g, i*
9	42	48	4	34	2	130	858	*a, b*
10	110	45	16	99	0	270	1186	*a, b, c, d, f, g, l, m*
11	30	33	9	95	1	168	703	*a, b, c, d, f*
12	75	54	6	53	5	193	1047	*a, b, c, d, f*
13	80	53	4	24	4	165	1018	*a, b, d*
14	20	28	0	4	0	52	833	*a, b, g*
15	6	1	0	0	0	7	152	*a, g, **
16	26	21	56	28	1	132	1529	*a, b, c, g*
17	2	19	37	29	0	87	1657	*a, b*
18	9	4	28	25	1	67	1721	*a, b, g*
Totals	829	778	714	980	64	3365	24540	

Supplementary Table 1 | Micromap FOVs. Data used in the analysis of chromatic micromaps, with numbers of cells in each color class (determined by preferred spatially uniform conditions). Figures showing data from a given FOV are listed in the 'Notes' column. ***a***: Fig. 3c. ***b***: Fig. 2d-f. ***c***: Fig. 4b,c; Supplementary Figs. 6-8 [five additional FOVs (not tabulated) were collected for these analyses of uniform vs bar stimuli, but not used for micromaps]. ***d***: Fig. 2g; Supplementary Fig. 3a-f. ***e***: Supplementary Fig. 3g-l. ***f***: Supplementary Fig. 9c,d. ***g***: Supplementary Fig. 5. ***h***: Fig. 3a,b. ***i***: Fig. 4d,e [two additional FOVs (not tabulated) were collected for this spatial analysis, but not used for micromaps]. ***j***: Fig. 1a-c. ***k***: Supplementary Fig. 10c. ***l***: Fig. 2a-c. ***m***: Fig. 4a; Supplementary Fig. 9a. *Omitted from other FOV-level analyses due to poor cell labeling.

Primary classification	Uniform n (%)
Red: +L, L-M, -M	383 (31.24)
Green: M, M-L, -L	186 (15.17)
Blue: +S, -L-M	300 (24.47)
Yellow: -S	357 (29.12)
Totals	1226 (100)

Alternative classification	Uniform n (%)	Bars n (%)
Top quadrant: 45 to 135 º	366 (29.85)	587 (47.88)
Bottom quadrant: 225 to 315 º	178 (14.52)	358 (29.2)
Right quadrant: -45 to 45 º	321 (26.18)	249 (20.31)
Left quadrant: 135 to 225 º	361 (29.45)	32 (2.61)
Totals	1226 (100)	1226 (100)

Supplementary Table 2 | Cell classification. Primary classification (left columns): number of cells in each class, based on preferred spatially uniform condition. Alternative classification (right columns): number of cells in each class, based on location of cells in the polar histograms of Supplementary Fig. 7, which accounts for both red/green and blue/yellow contributions to the cell's response.

Supplementary References

1. Landisman, C. E. & Ts'o, D. Y. Color processing in macaque striate cortex: relationships to ocular dominance, cytochrome oxidase, and orientation. *J. Neurophysiol.* **87**, 3126-37 (2002).
2. Conway, B. R. Spatial structure of cone inputs to color cells in alert macaque primary visual cortex (V-1). *J. Neurosci.* **21**, 2768-83 (2001).
