# Supplementary material for: Chromatic micromaps in primary visual cortex
Source: Nat Commun. 2021 Apr 19;12:2315. doi: 10.1038/s41467-021-22488-3 (PMC8055908; doi:10.1038/s41467-021-22488-3)
Supplement: Supplementary file 3 — Reporting Summary [file 41467_2021_22488_MOESM3_ESM.pdf]

nature research

Corresponding author(s): R. Clay Reid

Last updated by author(s): Feb 28, 2021

Reporting Summary

Nature Research wishes to improve the reproducibility of the work that we publish. This form provides structure for consistency and transparency in reporting. For further information on Nature Research policies, see our Editorial Policies and the Editorial Policy Checklist.

Statistics

For all statistical analyses, confirm that the following items are present in the figure legend, table legend, main text, or Methods section.

n/a	Confirmed	
☐	☒	The exact sample size (*n*) for each experimental group/condition, given as a discrete number and unit of measurement
☐	☒	A statement on whether measurements were taken from distinct samples or whether the same sample was measured repeatedly
☐	☒	The statistical test(s) used AND whether they are one- or two-sided<br>*Only common tests should be described solely by name; describe more complex techniques in the Methods section.*
☐	☒	A description of all covariates tested
☐	☒	A description of any assumptions or corrections, such as tests of normality and adjustment for multiple comparisons
☐	☒	A full description of the statistical parameters including central tendency (e.g. means) or other basic estimates (e.g. regression coefficient) AND variation (e.g. standard deviation) or associated estimates of uncertainty (e.g. confidence intervals)
☐	☒	For null hypothesis testing, the test statistic (e.g. F, t, r) with confidence intervals, effect sizes, degrees of freedom and P value noted<br>*Give P values as exact values whenever suitable.*
☒	☐	For Bayesian analysis, information on the choice of priors and Markov chain Monte Carlo settings
☒	☐	For hierarchical and complex designs, identification of the appropriate level for tests and full reporting of outcomes
☒	☐	Estimates of effect sizes (e.g. Cohen's *d*, Pearson's *r*), indicating how they were calculated

Our web collection on statistics for biologists contains articles on many of the points above.

Software and code

Policy information about availability of computer code

Data collection	MPScope 2.0
Data analysis	Matlab R14-R2017a; ImageJ

For manuscripts utilizing custom algorithms or software that are central to the research but not yet described in published literature, software must be made available to editors and reviewers. We strongly encourage code deposition in a community repository (e.g. GitHub). See the Nature Research guidelines for submitting code & software for further information.

Data

Policy information about availability of data

All manuscripts must include a data availability statement. This statement should provide the following information, where applicable:

- Accession codes, unique identifiers, or web links for publicly available datasets
- A list of figures that have associated raw data
- A description of any restrictions on data availability

Source data for Figs. 2c-f; 3c; 4b-f; and Supplementary Figs. 6a-c; 7a,c,e,g,i,k,m,o; 8a-c; and 9c-e are provided with this paper. Other data that support the findings of this study are available from the corresponding author upon reasonable request.

Field-specific reporting

Life sciences study design

All studies must disclose on these points even when the disclosure is negative.

Sample size	No statistical methods were used to determine sample size, but the numbers of animals and FOVs used in this study are consistent with other published work.
Data exclusions	All data exclusions are described in the text. From the Statistics and Reproducibility section: "Every FOV analyzed for color maps is listed in Supplementary Table 1, and none were excluded except for failed labeling with calcium indicator."
Replication	All attempts at replication were successful.
Randomization	No randomization was used.
Blinding	No explicit blinding was used. However, the experimenter was blind to which neurons/compartments (e.g., blobs) of V1 were labeled with calcium indicator, resulting in an unbiased sampling of neurons.

Reporting for specific materials, systems and methods

We require information from authors about some types of materials, experimental systems and methods used in many studies. Here, indicate whether each material, system or method listed is relevant to your study. If you are not sure if a list item applies to your research, read the appropriate section before selecting a response.

Materials & experimental systems

n/a	Involved in the study
☒	☐ Antibodies
☒	☐ Eukaryotic cell lines
☒	☐ Palaeontology and archaeology
☐	☒ Animals and other organisms
☒	☐ Human research participants
☒	☐ Clinical data
☒	☐ Dual use research of concern

Methods

n/a	Involved in the study
☒	☐ ChIP-seq
☒	☐ Flow cytometry
☒	☐ MRI-based neuroimaging

Animals and other organisms

Policy information about studies involving animals; ARRIVE guidelines recommended for reporting animal research

Laboratory animals	We used four adult macaque monkeys (Macaca mulatta, 3 males and 1 female, 8-12 years old, 9-18 kg) in this study.
Wild animals	No wild animals were used in this study.
Field-collected samples	No field-collected samples were used in this study.
Ethics oversight	All surgical and experimental procedures were in accordance with National Institutes of Health and United States Department of Agriculture guidelines and were approved by the Harvard Medical Area Standing Committee on Animals.

Note that full information on the approval of the study protocol must also be provided in the manuscript.
